# Neurological complications and death in children with dengue virus infection: report of two cases

**DOI:** 10.1186/s40409-017-0115-x

**Published:** 2017-04-27

**Authors:** Neydi Osnaya-Romero, Maria-Gabriela Perez-Guille, Sandra Andrade-García, Erika Gonzalez-Vargas, Rebeca Borgaro-Payro, Sandra Villagomez-Martinez, José de Jesús Ortega-Maldonado, Jose Luis Arredondo-García

**Affiliations:** 1 Intituto Nacional de Pediatria Insurgentes Sur 3700, letra C, Col. Insurgentes Cuicuilco, CP 04530 Ciudad de México, Mexico; 2 Morelense Children’s Hospital, Emiliano Zapata, Morelos Mexico

**Keywords:** Dengue, Dengue virus, Neurological disorders, Death, Children

## Abstract

**Background:**

Dengue virus infection can have different complications; the best known is hemorrhagic dengue fever. However, other effects such as neurological disorders may endanger the lives of patients. Dengue neurological manifestations can be confused with encephalitis symptoms and can lead to cerebral edema and death. Therefore, we consider important in the endemic areas to take into account the diagnosis of dengue encephalitis in patients with neurological disorders, and to request the determination of serology in cerebrospinal fluid for the NS1 antigen test.

**Case presentation:**

We present the cases of two patients from the state of Morelos, Mexico, with 17 and 14 years of age. Both cases presented a rapid evolution characterized by fever, seizures and neurological deterioration secondary to severe cerebral edema that evolved to cerebral death in both cases. The diagnosis of brain death was confirmed by electroencephalogram in both patients. The two patients were submitted to serology for NS1 that tested positive in both cases. They died between the second and fifth day after admission.

**Conclusions:**

Retrospective studies have found that up to 4% of the patients have dengue virus infections, which leads us to believe that in endemic areas, this infection should be suspected in cases of encephalic and febrile symptoms. RT-PCR should be performed to identify cases of encephalitis caused by the dengue virus, and early interventions should be performed to attempt to reduce the morbidity and mortality of these cases.

## Background

The WHO defines dengue as a mosquito-borne viral disease, whose global incidence has grown dramatically in recent decades. It is estimated that about half of the world’s population is now at risk. Dengue virus is transmitted by female mosquitoes, mainly *Aedes aegypti* and to a lesser extent by *A. albopictus*. These mosquitoes also transmit chikungunya fever, yellow fever, and Zika virus infection. Dengue is widespread in the tropics, with local variations in risk that are largely dependent on rainfall, temperature, and unplanned rapid urbanization.

Severe dengue (previously known as dengue hemorrhagic fever) affects most Asian and Latin American countries and has become one of the leading causes of hospitalization and death in children and adults in those regions [1].

Dengue virus fever can have deadly consequences. There are records of this disease since the Jin Dynasty (265–420 AD), and the first epidemics were reported in 1780. It was originally defined as “break-bone fever” due to the symptoms commonly observed in those afflicted.

Dengue virus (DENV) belongs to the family *Flaviviridae* and is an RNA virus composed of three structural protein genes, which encode the nucleocapsid or core (C) protein, a membrane-associated (M) protein, an enveloped (E) glycoprotein and seven non-structural (NS) proteins. Four serotypes have been described to date: DV-1, DV-2, DV-3, and DV-4 [2].

After being bitten by *A. aegypti* carrying the virus, individuals are infectious during the first five days of viremia. When an infected individual is bitten again by a mosquito, that mosquito will ingest infected blood; a blood meal involving another person thereby perpetuates the cycle. Isolating the virus requires serum collection during the first five days after symptoms appear [3].

All four serotypes have been identified in 29 states of Mexico, with a peak number of cases in 2007 and 2009. However, differences in records of suspected cases are caused by overreporting in endemic areas and underreporting in non-endemic areas. IgM antibodies, which are only present in 80% of patients during the febrile stage and can persist for three months, must be detected for diagnosis verification. In contrast, identification of IgG antibodies indicates a secondary infection. Polymerase chain reaction (PCR) detection of the NS1 antigen during the first five days of fever can indicate viral replication or an infection when other tests are negative.

Cytokine production facilitates capillary permeability, favoring fluid extravasation and mononuclear infiltration into muscles along with increases in creatine phosphokinase, producing myalgia. Treatment includes controlling fever and pain by physical means or with paracetamol [4].

DENV infection can have different clinical presentations, the most classic of which is a difficult-to-control fever accompanied by myalgia, arthralgia headache, retroocular pain, rash, and abdominal pain. Severe cases can present with epistaxis, petechiae, gastrointestinal (GI) bleeding, ascites, pleural effusion, heart attack, hypotension, tachycardia, alterations in state of consciousness, fulminant hepatitis, myocardiopathy, and encephalitis. Severe dengue can also cause shock and death [3]. In addition, there are reports that approximately 20% of cases of severe dengue develop vital organ involvement and complications [5].

Severe liver disorders are the most frequent complication, as diagnosed by significant alterations in liver function and other signs such as metabolic encephalopathy, jaundice, and alithiasic cholecystitis [6]. Cardiac conditions present with supraventricular bradycardia or tachycardia, T-wave inversion, and cardiac dysfunction [7]. Moreover, acute renal failure has been reported, associated with high mortality rates in 3% of dengue patients with shock [8]. Other complications involve aplastic anemia, rash, severe thrombocytopenia, pulmonary complications, cholecystitis, hemophagocytic syndrome, pancreatitis, and acute abdomen [9–11].

It is estimated that approximately 10% of dengue patients present with neurological disorders during or after infection, suggesting that the virus may induce neurological dysfunction, either directly by invading and infecting nervous tissue or indirectly via other organs that affect nervous function. These neurological complications include encephalitis/encephalopathy, meningitis, myelitis, cerebellitis, and acute disseminated encephalomyelitis. Up to 4% of dengue patients may develop neuromuscular complications such as myalgia, myositis, rhabdomyolysis and hypokalemic paralysis. Neurological complications frequently accompany severe dengue infection [12, 13].

Such potential neurological complications reaffirm the need to include neurological manifestations as a criterion of infection severity, as suggested by the WHO since 2009, because these manifestations can result in significant sequelae and even death [14, 15].

Here, we describe a case of two sisters in Axochiapan, Morelos, Mexico (a dengue endemic/epidemic region), who presented with fever and seizures caused by dengue and unfortunately died as a result of the infection.

Axochiapan is located in sanitary jurisdiction number 3 of the state of Morelos, geographically located at 18° 30″ north latitude and 98° 45″ west longitude of the Greenwich meridian, at a mean height of 1030 meters asl, with a surface area of 1423.10 km^2^. The 2010 census reported a population of 33,695, of which 60.3% are poor (48.7% moderate poverty and 11.6% extreme poverty); 31.9% of the people do not have access to health services, and 19.6% of the population live in poor-quality housing, do not have basic services, and lack access to adequate food [16, 17].

According to a WHO and PAHO report released in August 2016, there are 643,577 confirmed cases of dengue globally. In Mexico, 26,665 cases were reported with a lethality of 2.49; the serotypes identified include DN-1, -2, -3, and -4.  As of September 2015, Morelos Health Services had recorded 335 cases of dengue throughout the state, of which 34 cases occurred in the municipality of Axochiapan. In 2016, 515 cases were reported in the state of Morelos, of which 30 correspond to the municipality of Axochiapan, with a rate of 81.86 and including serotypes DV-1, −2, and −3 [18, 19].

## Case Presentation

### Case 1

**Fig. 1:**
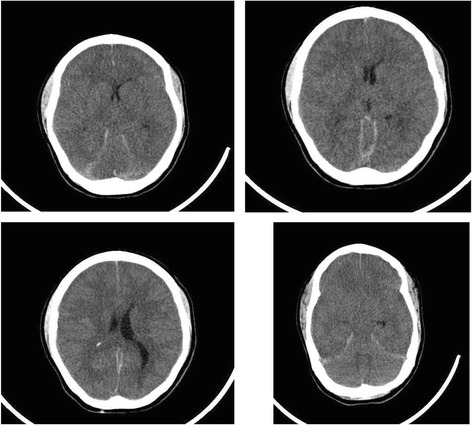
Computerized axial tomography of the brain with diagnosis of global cerebral edema, showing effacement of the subarachnoid cisterns and compression of the cerebral sulcus and gyrus and of the right ventricular system

The patient, a 17-year-old female, was admitted to the hospital on June 4, 2016, at 2:50 am. She had a history of seizures at eight years of age. She was managed with diphenylhydantoin for two years, after which the medication was discontinued. Her current illness began after returning from a school trip. At the end of the day, her teachers observed generalized tonic-clonic movements with increased muscle tone, though the duration was not noted. She was transferred to the regional hospital in Xochitepec, Morelos, where diphenylhydantoin was administered. However, her seizures continued. Epileptic status was suspected, and the decision was made to protect the airways with orotracheal intubation. She was then moved to Children’s Hospital of Morelos. On admission, she did not exhibit respiratory effort, corneal reflex, or cough reflex. ACT revealed cerebral edema and severe ventricular collapse (Fig. 1).

Her laboratory and blood tests reported are presented in Table 1. The patient remained on respiratory support. Two electroencephalograms (EEGs) were performed, with both being isoelectric. Based on the clinical and EEG reports, brain death was determined, and the patient was declared dead two days after admission. Reported serology for dengue NS1 via enzyme-linked immunoassay (ELISA) was positive; PCR to detect enterovirus in the cerebrospinal fluid (CSF) was negative.

**Table 1:** Laboratory tests reported in the patients studied

Parameter	Case 1 (17 years)	Case 2 (14 years)
Weight (kg)	52	45
Glucose mg/dL	109	148
Serum sodium meq/L	141	156
Serum potassium meq/L	2.2	3.29
Serum calcium mg/dL	6.9	6.8
Serum creatinine mg/dL	0.6	0.7
Total bilirubin mg/dL	0.28	0.30
Direct bilirubin mg/dL	0.10	0.11
Indirect bilirubin mg/dL	0.16	0.19
Glutamic pyruvic transaminase U/L	33	25
Glutamic oxaloacetic transaminase U/L	23	17
Alkaline phosphatase U/L	64	88
Phosphorus mg/dL		3.21
Prothrombin time sec.	15.9	15.7
Thromboplastin time sec.	26.5	24.2
INR	74.21	1.33
Hemoglobin g/dL	12	10.6
Hematocrit %	34.1	32.3
Platelets × 10^3^/mm^3^	152	143
Leucocytes mm^3^	4.54	5.93
Segmented %	85	94
Bands %		4
Monocytes %	2	1
Lymphocytes %	13	5
Fibrinogen mg/dL		272
CSF		
Proteins (mg/dL)	1629.8	84.2
Glucose mg/dL	1	87
Chloride mEq/L	134	122
Leucocytes mm^3^	0	0
Gram stain	Negative	Negative
CSF culture	Negative	Negative
Ant doping	Negative	Negative
IgA (mg/dL)	148	150
IgG (mg/dL)	750	867
IgM (mg/dL)	117	123
Immunoglobulin E (UI/mL)	87.1	327

### Case 2

**Fig. 2:**
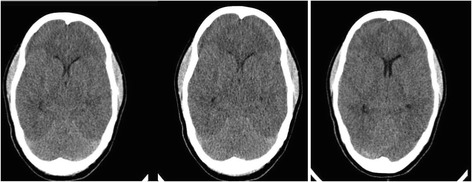
Computerized axial tomography with diffuse cerebral edema, showing loss of differentiation between grey and white matter and compression of the ventricles

A 14-year-old female was admitted to the hospital on June 5, 2016, at 2:20 am. The patient’s symptoms started 12 h after clinical manifestation in her sister (Case 1). She had a 39 °C fever and headache progression for 24 h. She presented with psychomotor agitation, followed by generalized tonic-clonic seizures. She was taken to Axochiapan Morelos Hospital for assessment. On admission, she exhibited neurological deterioration requiring orotracheal intubation and was sent to Children’s Hospital of Morelos, where cranial computerized axial tomography (CAT) revealed severe cerebral edema and uncal herniation (Fig. 2). Her laboratory blood tests are presented in Table 1. She was transferred to the intensive care unit where hemodynamic and antiedema care was provided. On beginning intensive care, clinical data indicated brain death, which was confirmed by three isoelectric EEGs. The patient died in the hospital five days after admission. Serology positive for dengue NS1 by ELISA was reported, and PCR was performed to detect enterovirus in the CSF, with negative results.

Both patients underwent anti-doping and immunoglobulin testing on admission (Table 1). At the time of interview, family members responsible for both patients were asked about any background that might indicate probable toxic poisoning of the patients, but no positive indications were obtained. When questioned about whether there was another member of the family with the same clinical picture, the mother of the children said that on June 3 of the same year, her 6-year-old daughter had a fever of 38 °C. She was taken to a health center in the community of Axochiapan Morelos where paracetamol was prescribed, which improved the symptoms, and no other complications presented. Due to the history of the complications suffered by the patients, serology for dengue NS1 in the 6-year-old child was performed by ELISA, with a positive result.

In our country, the ELISA and PCR tests used for the detection of dengue virus are based on the Epidemiological Diagnostic and Reference guidelines of the Department of Health [20], performed as follows.

### Platelia NS1 ELISA

A commercial kit (Platelia NS1)® that is an immunoenzymatic method useful for qualitative or semiquantitative detection of the dengue virus NS1 antigen in human serum or plasma was used. The assay utilizes murine monoclonal antibodies (mAbs) for antigen capture and detection. Samples from patients and controls are incubated directly and simultaneously with the diluted conjugate for 90 min at 37 °C. Any NS1 antigen present in the sample will form an immune complexes, mAb-NS1-mAb/peroxidase, which is detected using the enzymatic development solution, promoting a colored reaction (blue). After 30 min of incubation at room temperature, the enzymatic reaction is stopped by the addition of an acid solution (stop solution). The optical density obtained at 450/620 nm is proportional to the amount of NS1 antigen present in the sample. The presence of the antigen in a sample is established by comparing the optical density of this sample with that obtained in threshold serum. The results are interpreted according to the following criteria:

- **Nonreactive**: A sample with an index value < 0.5 is considered non-reactive for the dengue virus NS1 antigen.
- **Equivocal**: A sample with an index value between 0.5 and 1.0 is considered equivocal for the dengue virus NS1 antigen; such a sample should be analyzed again.
- **Reactive**: A sample with an index value >1.0 is considered reactive for the dengue virus NS1 antigen.

### Detection and identification of dengue virus serotypes by multiplex real-time RT-PCR

Multiplex real-time reverse transcriptase-PCR (RT-PCR) has the ability to monitor the progress of amplification in each cycle. Data are collected from the first reaction cycle to the last, allowing relative and absolute quantification of the viral load present in the sample. Amplification of a specific sequence is monitored by detection of fluorescence signals emitted during the PCR cycles. The presence of high concentrations of RNA or number of copies of genetic material will cause an early increase in fluorescence, whereas low concentrations of RNA will cause an increase in fluorescence in later reaction cycles. As the dengue virus circulates in detectable concentrations during the acute phase of the disease (0–5 days), this assay is only used during the acute phase and in samples positive for the NS1 antigen. The results are interpreted according to the following criteria:

- **Negative**: There is no viral RNA present, or there is an extremely low viral load related to the sensitivity of the method.
- **Positive**: “Dengue virus infection is only indicated by NS1 positivity.”

A positive result is indicative of recent infection and the presence of viral RNA of the serotype(s) detected. Multiplex real-time RT-PCR determines the presence of RNA from the four dengue virus serotypes in serum and plasma. A positive result is indicative of recent infection caused by the serotype(s) identified.

## Discussion

Both patients had seizures and alterations in the state of consciousness. Toxic poisoning was ruled out by the clinical history and a negative anti-doping test. None of the CBC results pointed to a bacterial process. CSF was analyzed by gram-staining to assess the presence of bacteria and by RT-PCR to detect the presence of enterovirus, with negative results in both cases. Proteins, which may be present or not in cases of encephalitis, were found in the CSF in only one patient [21]. Therefore, the most likely diagnosis to be considered was encephalitis, probably caused by dengue virus infection based on the endemic area where the patients lived; this is why a determination by ELISA was requested. Having obtained a positive dengue virus NS1 antigen ELISA, with a sensitivity of 80–100% and a specificity of 94–100%, and a negative enterovirus PCR, with a sensitivity of 100% and a specificity of 77%, the diagnosis of neuro-dengue was confirmed in both patients [20]. The clinical manifestations presented by these patients coincide with those reported by Carod-Artal et al. [12], who concluded that the diagnosis of neuro-dengue can be established in patients with fever and neurological manifestations in dengue endemic regions.

Neurological manifestations in patients with DENV infection are not common. Indeed, some authors have reported such manifestations in only 5.4 to 10% of patients with severe dengue. These manifestations of the central nervous system appear as headache, vomiting, and neck rigidity. Only when performing specific serology for dengue can this neurological complication be identified; otherwise, cases are diagnosed as encephalitis or meningitis [12, 13]. Cases of dengue fever with neurological manifestations have a higher risk of death, as reported in studies in Brazil, with a mortality rate of 1.9% [22, 23]. When dealing with a febrile clinical picture, a systemic inflammatory response should be ruled out, as should other leptospirosis pathologies, malaria, infectious hepatitis, yellow fever, meningococcemia, rubella, and influenza [23]. It is not yet clear how damage is caused in such neurological alterations in dengue virus infection; the effect may be due to the virus infecting nerve cells or as a result of the disruption of other organs, such as the liver [14]. There are three ways in which neurological alterations occur in DENV infection: non-specific encephalopathy, specific encephalopathy, and post-infection encephalitis. Other explanations of neurological disorders include liver disease, cerebral edema, and hyponatremia, without discarding the possibility of direct damage by the virus to the central nervous system [24, 25].

Research on the etiopathology of neurological manifestations in dengue is diverse. For example, it was initially thought that neurological manifestations were due to anti-dengue antibodies [26, 27]. Later research reported that encephalitis occurs as a consequence of direct invasion of DENV into the brain, as viral antigens have been detected in the inferior olivary nucleus of the medulla oblongata and in the granular layer of the cerebellum [14].

In patients with suspected dengue virus infection and neurological manifestations, it is vital to document these alterations by means of imaging studies such as magnetic resonance imaging (MRI). However, given that this technique is not readily available in many health centers, a brain CAT scan could be used instead; if the patient dies, autopsy would provide more information about the alterations caused by this infection.

In the patients reported herein, electrolytes and liver function tests did not show alterations that could explain the neurological manifestations. A limitation of this study was that the only imaging technique available was a brain CAT scan, based on which the presence of severe cerebral edema was established. EEGs were also obtained, with total absence of EEG activity suggesting brain death. Unfortunately, autopsy could not be performed in these cases.

## Conclusion

To date, the physiopathology of neurological manifestations in DENV infection, as well as its incidence, are unknown. In some cases, patients are admitted with a diagnosis of encephalitis. Furthermore, retrospective studies have found that up to 4% of these patients have DENV infections, which leads us to believe that in endemic areas, dengue virus infection should be suspected in cases of encephalic and febrile symptoms. RT-PCR should be performed to identify cases of encephalitis caused by the dengue virus, and early interventions should be performed to attempt to reduce the morbidity and mortality of these cases.

## Abbreviations

CAT: Computerized axial tomography
CSF: Cerebrospinal fluid
CSF: Cerebrospinal fluid
DENV: Dengue virus
EEG: Electroencephalogram
ELISA: Enzyme-linked immunoassay
GI: Gastrointestinal
MRI: Magnetic resonance imaging
PCR: Polymerase chain reaction
RT-PCR: Reverse transcriptase polymerase chain reaction
